# Mapping the neuropathic pain biomarker landscape (2004–2024): A bibliometric analysis of thematic evolution, research silos, and the translational gap

**DOI:** 10.1097/MD.0000000000044265

**Published:** 2025-09-05

**Authors:** Zihao Zhang, Qingpei Hao, Gaoquan Lv, Shijun Peng, Tao Wang, Xin Chang, Yuepeng Wang, Jia Ouyang, Ruen Liu

**Affiliations:** a Department of Neurosurgery, Peking University People’s Hospital, Beijing, China; b Departments of Radiology, Peking University People’s Hospital, Beijing, China.

**Keywords:** bibliometric analysis, biomarker, neurofilament light chain, neuroimaging, neuropathic pain, translational gap

## Abstract

**Background:**

Despite a surge in neuropathic pain (NP) biomarker research over the past 2 decades, the translation of discoveries into clinical practice remains slow. To understand this translational gap, we conducted a comprehensive bibliometric analysis to map the field’s evolution, intellectual structure, and strategic challenges.

**Methods:**

We conducted a bibliometric analysis of NP biomarker-related publications from 2004 to 2024 using the Web of Science Core Collection (WoSCC) database. Tools including CiteSpace, VOSviewer and Scimago Graphica were employed to evaluate authors, institutions, countries/regions, journals, keywords and co-citations.

**Results:**

A total of 2437 articles were included in this study. The United States and European countries play a leading role, while China demonstrates high publication output but comparatively lower citation impact. Keyword analysis identified 5 major research clusters, exposing a clear thematic evolution from foundational “molecular mechanisms” towards technology-driven frontiers, including “neuroimaging” and emerging biomarkers like “neurofilament light chain” (NfL).

**Conclusion:**

This study provides a strategic map of the NP biomarker field, highlighting a persistent gap between robust basic science discovery and its clinical application. The field’s fragmentation into distinct research “silos” (e.g., molecular, neuroimaging) underscores that the primary future challenge is enhancing interdisciplinary integration. Accelerating progress will depend on building bridges between these domains to develop the multi-modal biomarker strategies essential for improving patient care.

## 1. Introduction

Neuropathic pain (NP) is a debilitating chronic condition that arises from damage to the somatosensory nervous system, affecting an estimated 7% to 10% of the general population.^[1,2]^ Its complex origins, involving both the peripheral and central nervous systems, result in a wide array of challenging symptoms. Consequently, biomarkers have emerged as a critical tool with the potential to untangle this complexity, offering pathways to more accurate diagnosis, prognostic assessment, and personalized treatment strategies.^[3,4]^

Reflecting this promise, the past 2 decades have witnessed an explosion of research dedicated to discovering and validating NP biomarkers. However, this rapid growth in publications has not translated into widespread clinical success. The journey of biomarkers from laboratory discovery to routine clinical application remains notoriously slow and fraught with challenges, leaving a significant gap between research output and patient benefit. The field now faces a critical question: with thousands of studies published, what are the dominant research themes, where are the collaborative and intellectual bottlenecks, and which emerging areas hold the most promise?

To address this gap, this study performs a comprehensive bibliometric analysis of NP biomarker literature published from 2004 to 2024. Using analytical tools such as CiteSpace and VOSviewer, we systematically map the intellectual landscape of the field. Our objective is to identify the key research clusters, trace their evolution over time, highlight the most influential contributors and publications, and pinpoint emerging trends that are shaping the future of NP biomarker research. Ultimately, this analysis provides a data-driven “roadmap” of the NP biomarker field. By synthesizing 20 years of research, we offer a strategic overview that can help researchers identify critical knowledge gaps, foster more targeted collaborations, and accelerate the development of clinically meaningful biomarkers that can truly improve the management of NP.

## 2. Materials and methods

### 2.1. Data source and search strategy

**Figure 1.**
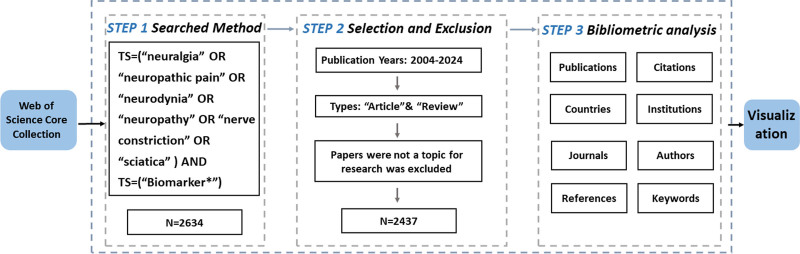
Detailed flowchart steps of the search strategy.

Publications were collected from the Web of Science Core Collection (WoSCC) database. Figure 1 illustrates the specific data retrieval techniques and inclusion criteria applied in this study. A systematic literature search was performed on October 28, 2024, to collect publications spanning from January 1, 2004, to the search date. English articles and reviews were included in the manuscript.

### 2.2. Bibliometric analysis and visualization

The title, authors, year of publication, country/region, institution, keywords, citations, abstracts, and references were extracted from the WoSCC database, with the files downloaded in a plain text format. The data were processed in Microsoft Excel 2019 to organize and perform preliminary analysis, including a polynomial regression model to estimate the number of publications for 2024. Collaboration networks among authors, institutions, countries/regions, journals, keyword clustering and co-cited reference was analyzed using VOSviewer (version 1.6.10) and Scimago Graphica (version 1.0.45). Keyword timeline mapping and construction of the dual-map overlay for journals were conducted using CiteSpace (version 6.4.R1, Chaomei Chen, Drexel University). CiteSpace parameters were set as follows: link retaining factor (LRF = 3), e for top N (e = 1.0), time span (2004–2024), years per slice (1), look back years (LBY = 5), links (strength: cosine, scope: within slices), selection criteria (g-index: k = 25), and minimum duration (MD = 1).

## 3. Results

### 3.1. Annual growth trend of publications and citations

**Figure 2.**
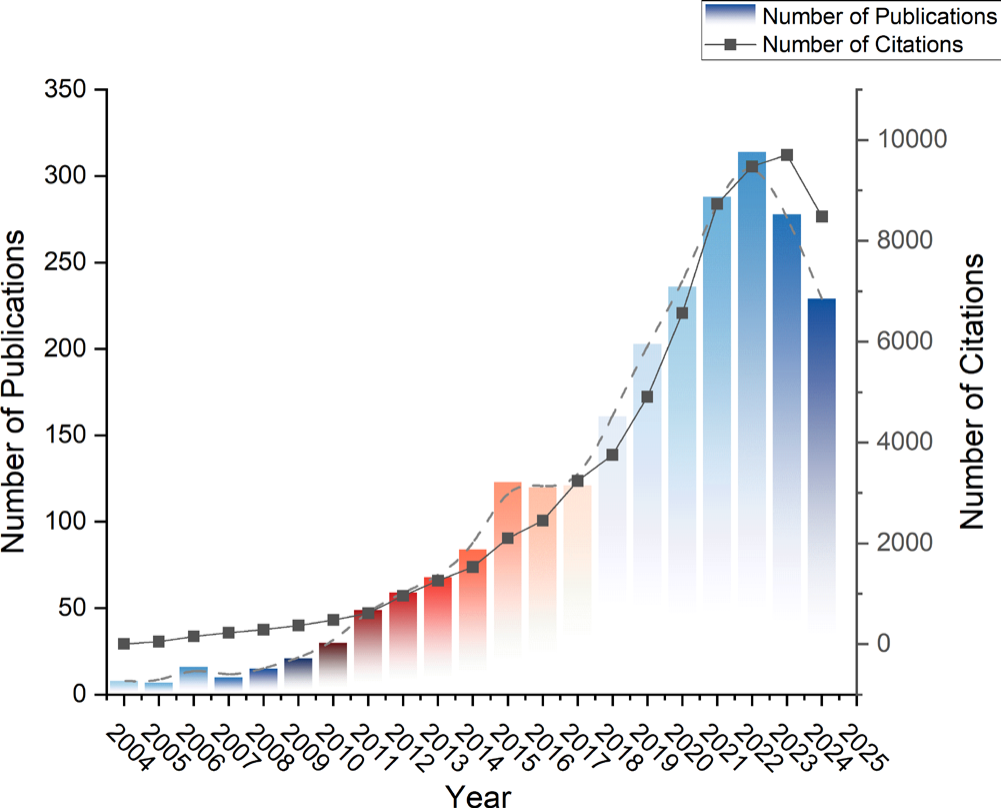
The annual number of publications and citations on NP biomarker from 2004 to 2024. NP = neuropathic pain.

**Figure 3.**
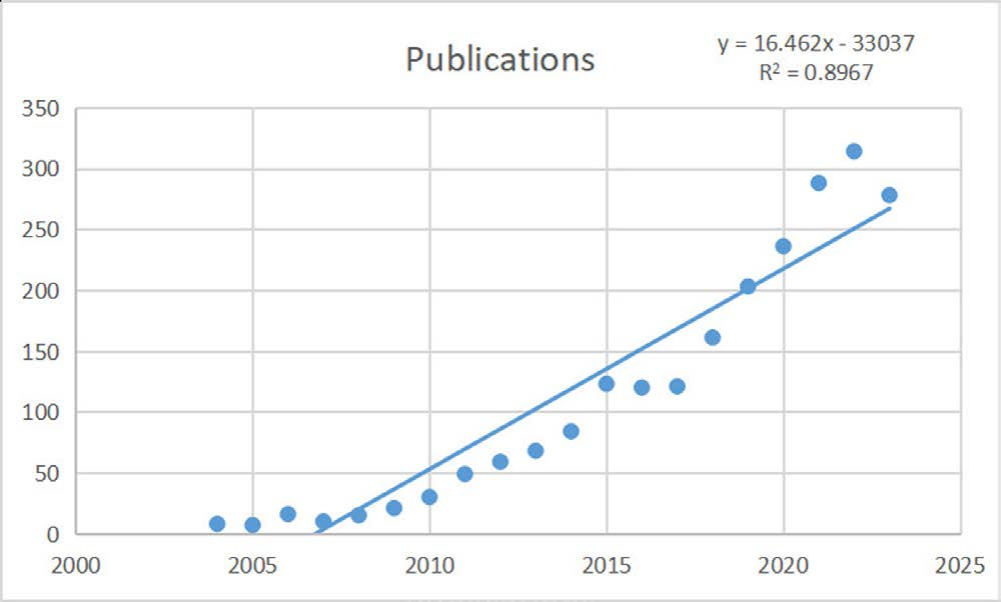
Regression analysis of annual publication trends on NP biomarker research. NP = neuropathic pain.

The final analysis included 2437 publications. The annual publication output showed a consistent growth pattern, rising steadily after 2009 and reaching a peak of 314 articles in 2022 (Fig. 2). A polynomial regression model confirmed a strong positive correlation between publication year and the number of articles (R² = 0.8967), with a projected total of 282 publications for the full year of 2024 (Fig. 3). Annual citations followed a similar upward trajectory, reflecting the field’s expanding influence (Fig. 2).

### 3.2. Authors and co-cited authors

**Figure 4.**
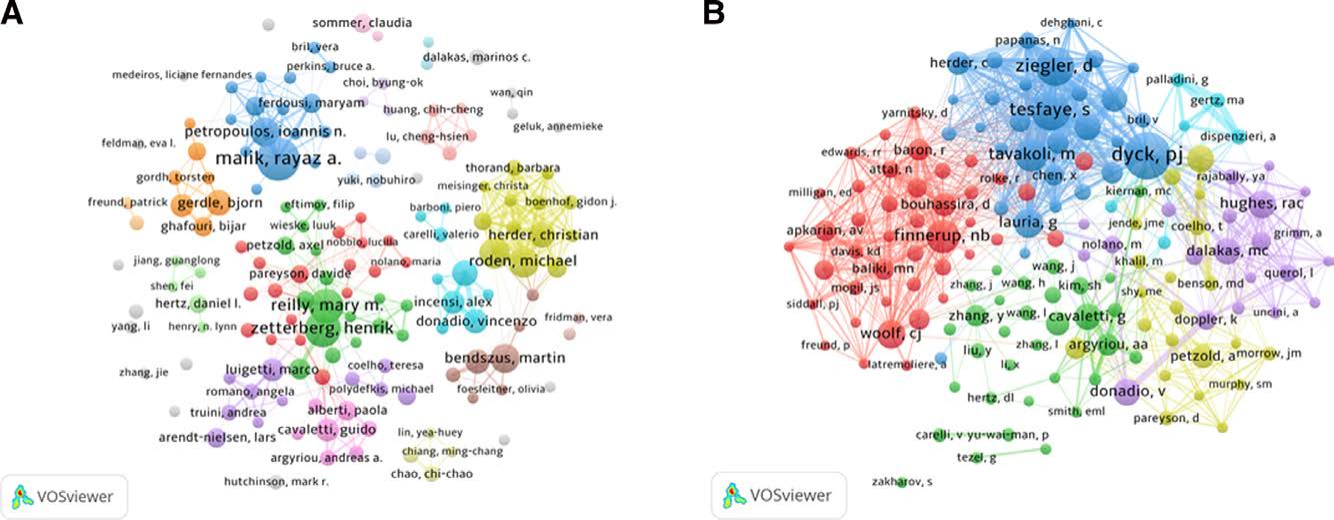
A network map showing authors (A) and co-cited authors (B) related to NP biomarker research. NP = neuropathic pain.

Over 14,000 researchers contributed to the field during the analyzed period. The most productive authors were Rayaz A. Malik (26 publications), Henrik Zetterberg (21 publications), and Mary M. Reilly (20 publications). In the co-citation analysis, Peter J. Dyck ranked as the most influential author with 255 co-citations, followed by Solomon Tesfaye (201) and Dan Ziegler (198) (Table 1). The co-authorship network reveals that the most prolific authors typically form the central nodes within large collaborative clusters (Fig. 4). Notably, the list of top 10 authors and co-cited authors is dominated by researchers from European countries, with a significant underrepresentation from Asian countries, including China (Table 1).

**Table 1:** Top 10 prolific authors and co-cited authors.

Rank	Author	Country	Count	Co-cited Author	Country	Count
1	Rayaz A Malik	UK	26	Peter J Dyck	USA	255
2	Henrik Zetterberg	Sweden	21	Solomon Tesfaye	UK	201
3	Mary M Reilly	UK	20	Dan Ziegler	Germany	198
4	Michael Roden	Germany	17	Mitra Tavakoli	UK	165
5	Dan Ziegler	Germany	17	Nanna Brix Finnerup	Denmark	165
6	loannis N Petropoulos	Qatar	16	Giuseppe Lauria	Italy	141
7	Martin Bendszus	Germany	16	Clifford J Woolf	USA	137
8	Christian Herder	Germany	14	Richard A C Hughes	UK	133
9	Rocco Liguori	Italy	14	David J Adams	Australia	129
10	Bjorn Gerdle	Sweden	14	Vincenzo Donadio	Italy	128

### 3.3. Analysis of the contributions of countries/regions

**Figure 5.**
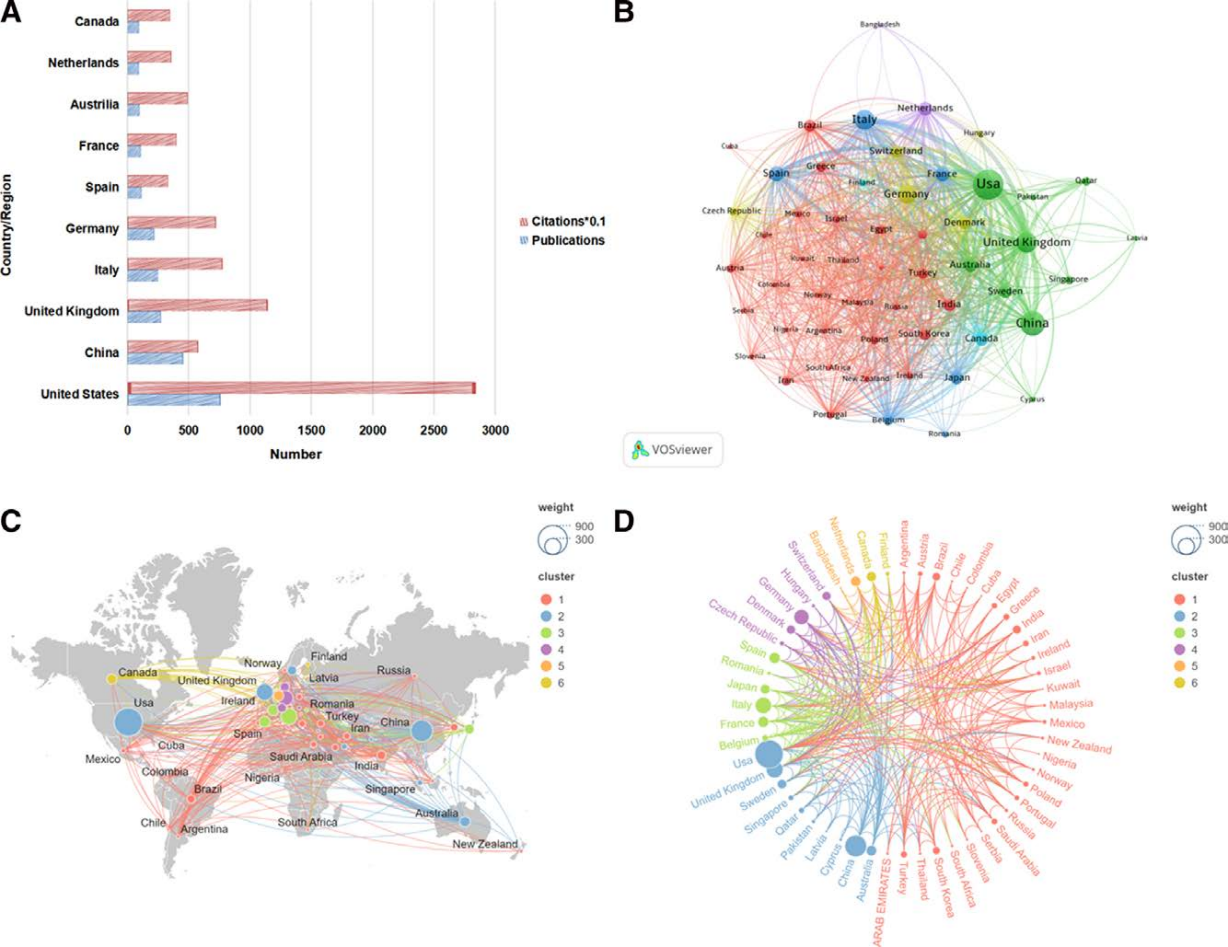
Analysis of countries/regions engaged in NP biomarker research. (A) The top 10 countries/regions publications and their citations. (B) A network visualization map showing countries/regions. (C) The global distribution of countries/regions in terms of publications. (D) A collaborative network map of institutions.

A total of 95 countries/regions contributed publications to NP biomarker research. The United States led in overall output with 761 publications and 28,430 citations, followed by China (454 publications; 5773 citations) and the United Kingdom (272 publications; 11,473 citations) (Table 2). The international co-authorship network reveals a dense collaboration core primarily among North American and Western European nations. The United States, the UK, and Germany form the most prominent and interconnected collaborative triangle, indicating strong research ties between these countries (Fig. 5).

**Table 2:** Top 10 productive countries/regions.

Rank	Country/Region	Publications	Citations
1	United States	761	28,430
2	China	454	5773
3	United Kingdom	272	11,473
4	Italy	255	7777
5	Germany	218	7242
6	Spain	119	3333
7	France	116	4038
8	Australia	103	4947
9	Netherlands	100	3576
10	Canada	95	3495

### 3.4. Analysis of the contributions by institutions

**Figure 6.**
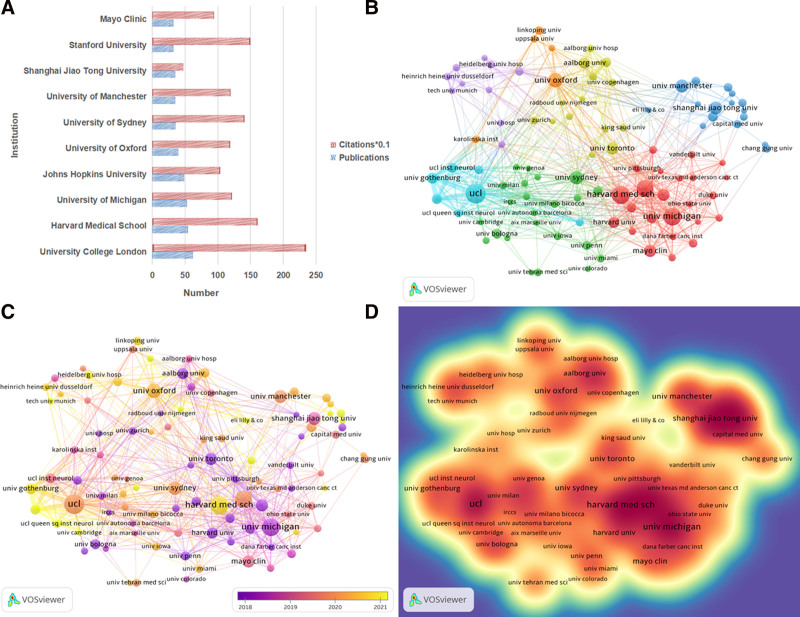
Analysis of institutions involved in NP biomarker research. (A) The top 10 institutions. (B) A network visualization map of institutions. (C) A overlay visualzation map. (D) A density network map. NP = neuropathic pain.

Analysis of institutional contributions identified 3767 participating institutions. University College London was the most productive institution, with 62 publications and 2348 citations, followed by Harvard Medical School (54 publications; 1610 citations) and the University of Michigan (22 publications; 726 citations) (Table 3). Shanghai Jiao Tong University was the only Asian institution among the top 10, ranking eighth by publication volume and tenth by total citations. The institutional co-authorship analysis revealed 7 major research clusters, with the largest clusters dominated by closely collaborating institutions from the UK and the United States (Fig. 6).

**Table 3:** The top 10 productive institutions.

Rank	Institution	Country	Publications	Citations
1	University College London	United Kingdom	62	2348
2	Harvard Medical School	United States	54	1610
3	University of Michigan	United States	53	1216
4	Johns Hopkins University	United States	49	1034
5	University of Oxford	United Kingdom	40	1187
6	University of Sydney	Austrilia	36	1408
7	University of Manchester	United Kingdom	35	1198
8	Shanghai Jiao Tong University	China	35	475
9	Stanford University	United States	32	1496
10	Mayo Clinic	United States	32	943

### 3.5. Analysis of the contributions of journals

**Figure 7.**
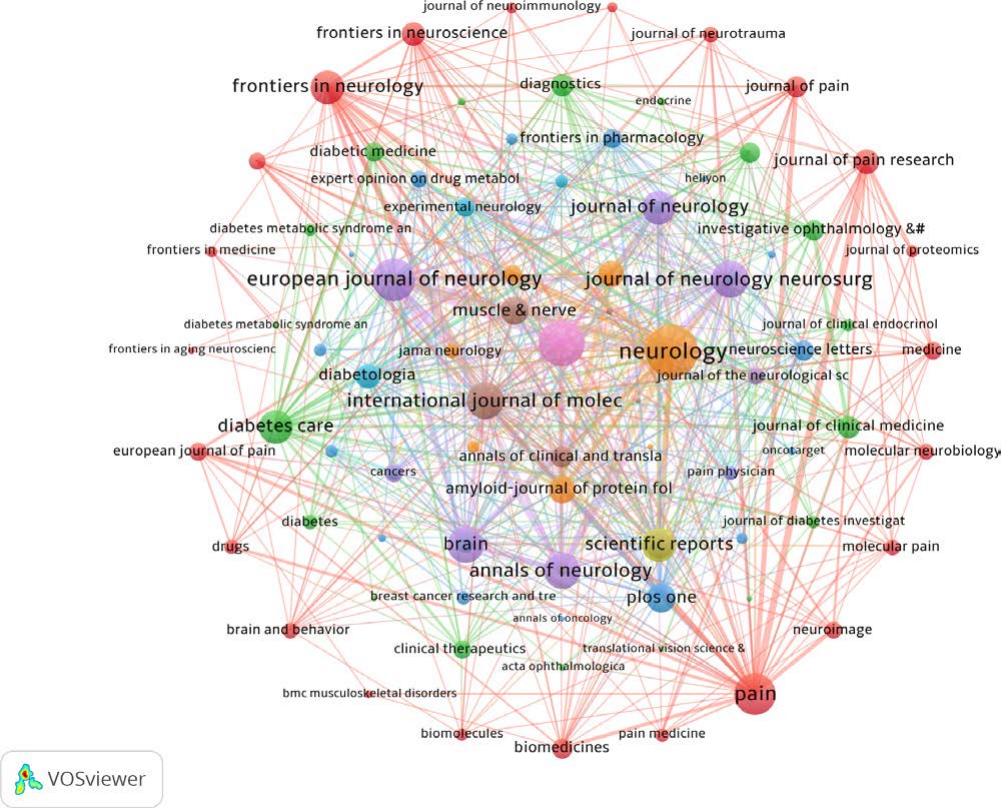
A network visualization map showing academic journals publishing research on NP biomarker. NP = neuropathic pain.

**Figure 8.**
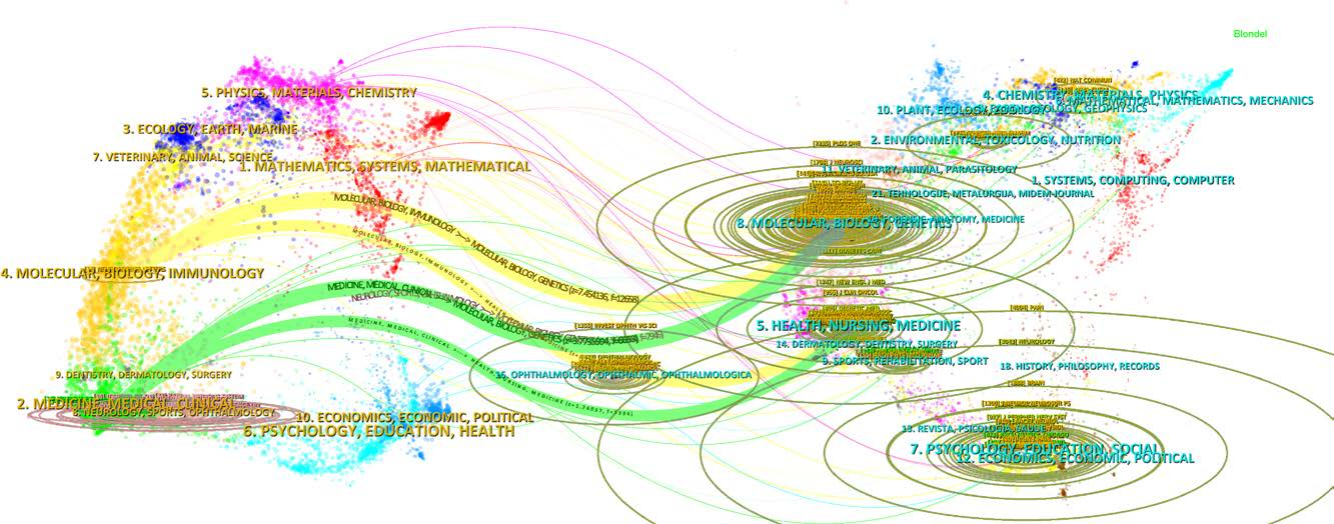
A dual-map overlay of journals related to research on NP biomarker. NP = neuropathic pain.

The analyzed articles were published across 896 journals. International Journal of Molecular Science (56 articles) and PLOS ONE (43 articles) were the most productive journals in the field (Table 4). Notably, most of the top 10 journals are classified in the highest quartiles (Q1/Q2) by Journal Citation Reports. As shown in Figure 7, there were positive citation relationships between different journals. The dual-map overlay revealed a distinct citation flow from basic science to clinical research; publications in “Molecular, Biology, Immunology” were heavily cited by articles from “Medicine, Medical, Clinical” journals, highlighting a strong translational research pathway (Fig. 8).

**Table 4:** The top 10 most productive journals.

Rank	Journal	JCR	Country	IF (2023)	Publications	Citations
1	International Journal of Molecular Science	Q1	Switzerland	4.9	56	901
2	PLOS ONE	Q1	United States	2.9	43	994
3	Pain	Q1	Netherlands	5.9	38	4227
4	Journal of the Peripheral Nervous System	Q1	United States	3.9	36	638
5	Scientific Reports	Q1	United Kingdom	3.8	35	578
6	Frontiers in Neurology	Q2	Switzerland	2.7	34	470
7	European Journal of Neurology	Q1	United Kingdom	4.5	30	435
8	Musle and Nerve	Q2	United States	2.8	24	413
9	Neurology	Q1	United States	7.7	23	1748
10	Brain	Q1	United Kingdom	10.6	21	1179

### 3.6. Analysis of keywords

**Figure 9.**
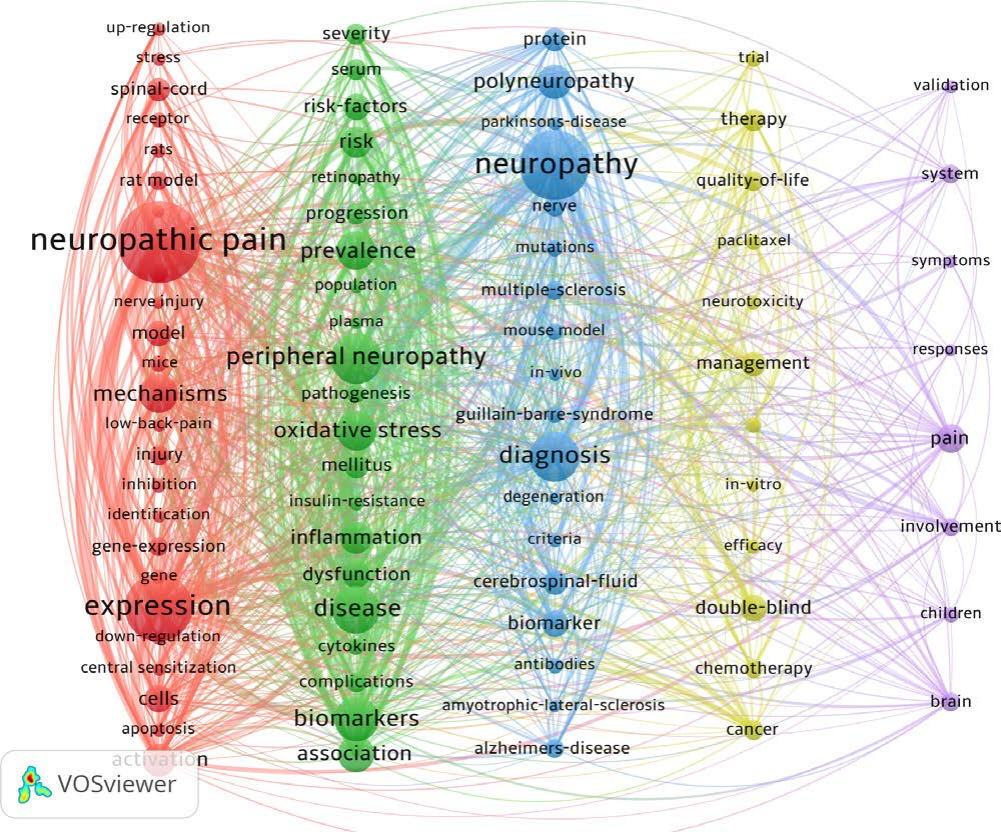
A network visualization map of keywords using VOSviewer.

**Figure 10.**
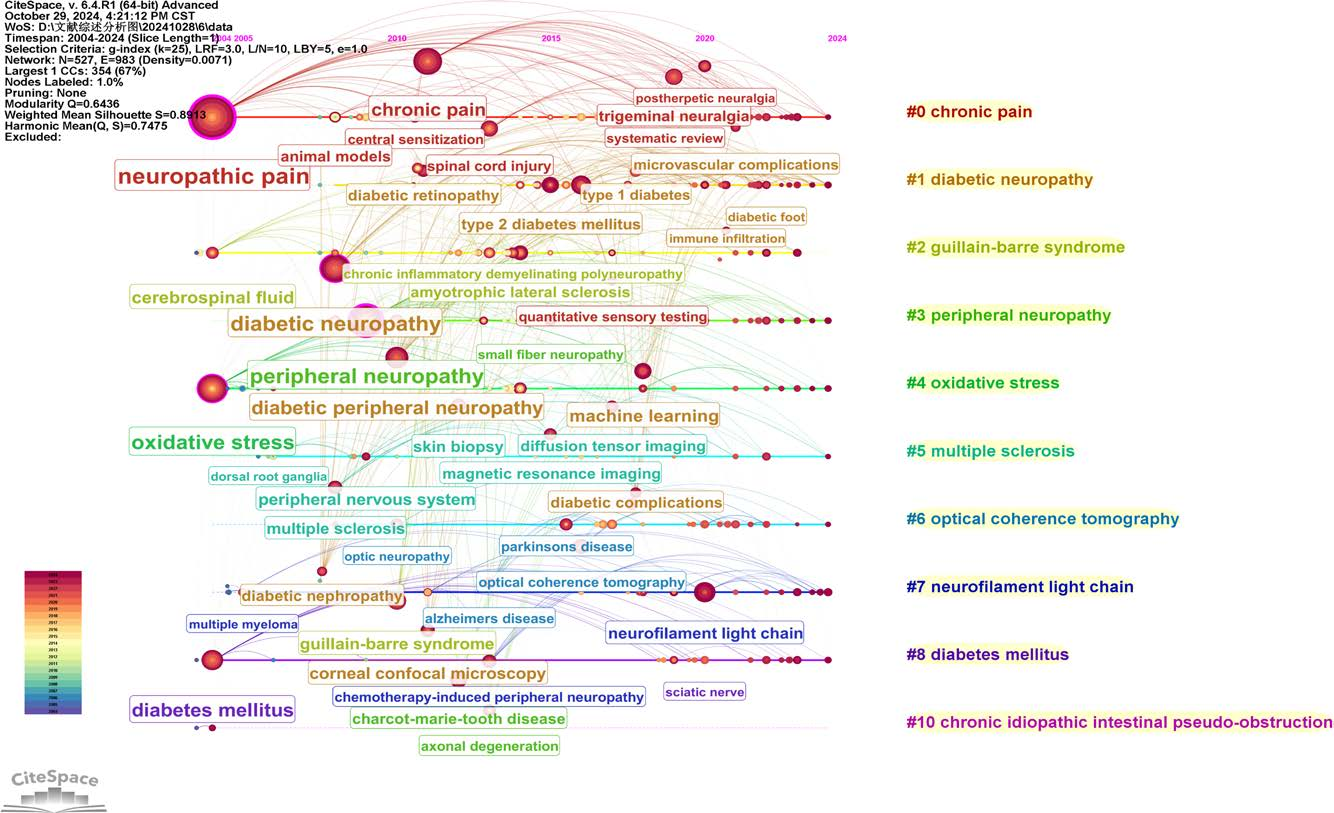
A timeline and keyword clustering display for the NP biomarker. NP = neuropathic pain.

**Figure 11.**
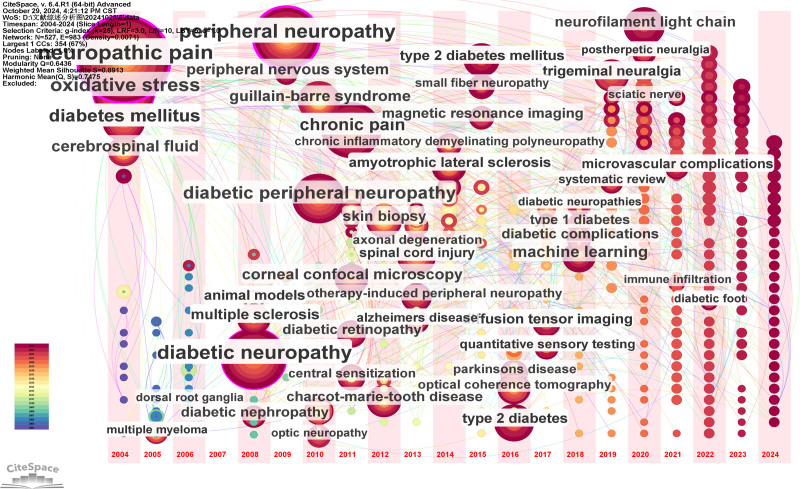
Keywords timezone view of NP biomarker. NP = neuropathic pain.

**Figure 12.**
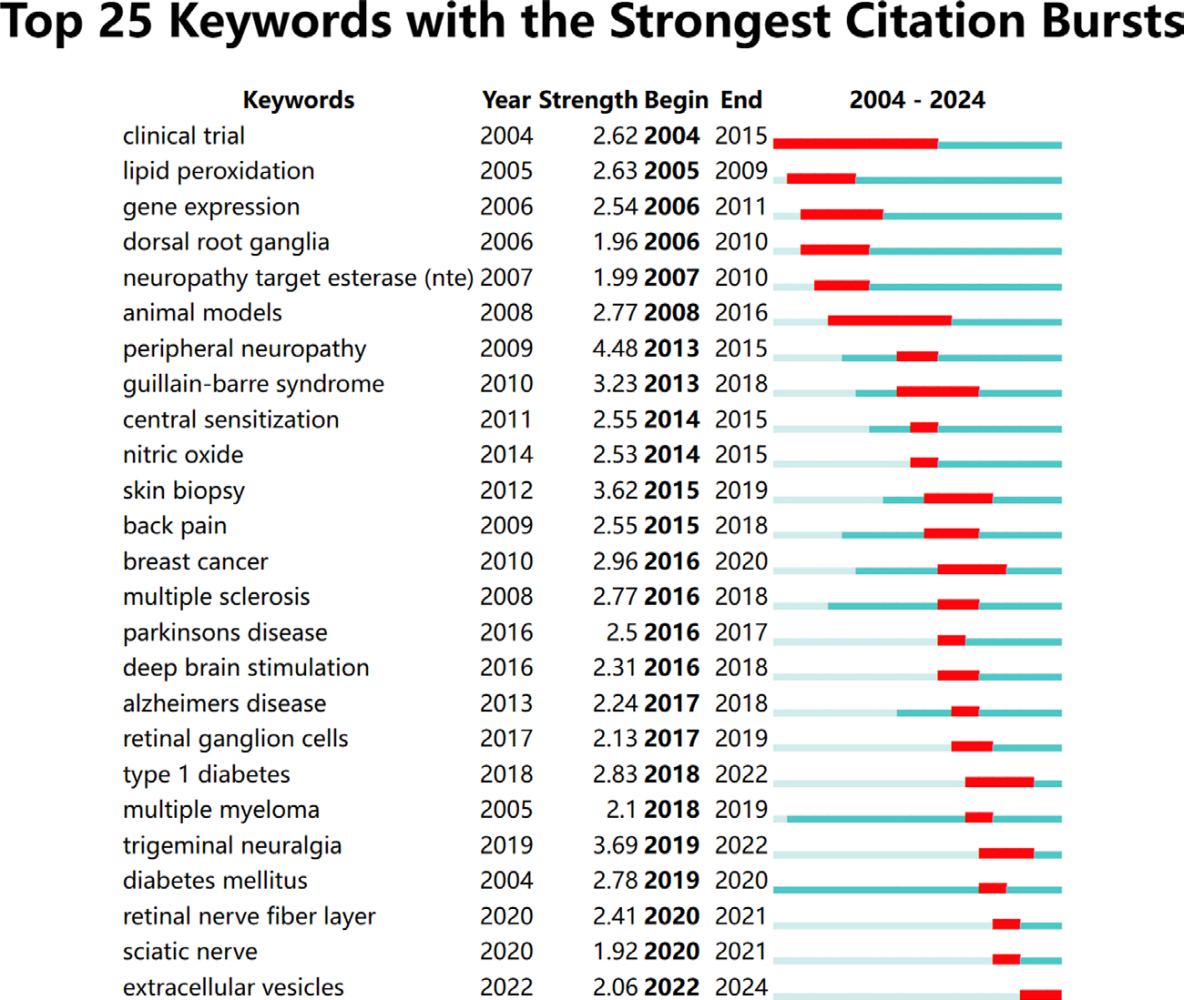
The top 25 keywords with the strong citation bursts in articles.

Co-occurrence analysis identified 84 high-frequency keywords that formed 5 major research clusters (Fig. 9). “NP” and “diabetic peripheral neuropathy” were the most persistent hotspots, remaining active throughout the entire 2004 to 2024 period (Figs. 10 and 11). Citation burst analysis pinpointed several emerging research fronts. Keywords with the strongest citation bursts since 2019 included “extracellular vesicles” (strength: 2.06, 2022–2024), and “trigeminal neuralgia” (strength: 3.69, 2019–2022) (Fig. 12). Other recently prominent keywords include “immune infiltration,” “diffusion tensor imaging (DTI),” and “neurofilament light chain.”

### 3.7. Analysis of highly-cited reference

**Figure 13.**
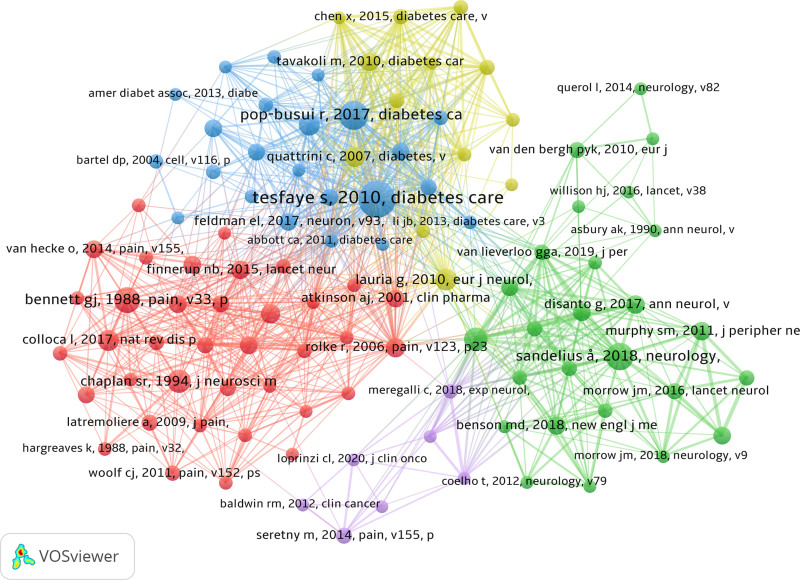
A network visualization map of the top 106 co-cited reference.

The 2437 source articles contained a total of 114,392 cited references. The co-citation analysis identified the 2010 Diabetes Care article, “Diabetic neuropathies: update on definitions, diagnostic criteria, estimation of severity, and treatments,” as the single most co-cited work (Table 5). Overall, 106 references were co-cited more than 20 times, forming the foundational knowledge base of the NP biomarker field (Fig. 13).

**Table 5:** The top 10 co-cited references involved in research on neuropathic pain biomarker.

Year	Title	Journal	Citation
2010	Diabetic neuropathies: update on definitions, diagnostic criteria, estimation of severity, and treatments	Diabetes Care	93
2017	Diabetic Neuropathy: A Position Statement by the American Diabetes Association	Diabetes Care	65
2018	Plasma neurofilament light chain concentration in the inherited peripheral neuropathies	Neurology	59
2018	Neurofilaments as biomarkers in neurological disorders	Nature Reviews Neurology	49
2010	European Federation of Neurological Societies/Peripheral Nerve Society Guideline on the use of skin biopsy in the diagnosis of small fiber neuropathy.	European Journal of Neurology	43
2005	Vascular risk factors and diabetic neuropathy	New England Journal of Medicine	40
2017	Neuropathic pain	Nature Reviews Disease Primers	40
2007	Surrogate markers of small fiber damage in human diabetic neuropathy	Diabetes	39
2006	Quantitative sensory testing in the German Research Network on Neuropathic Pain (DFNS): standardized protocol and reference values	Pain	37
2008	Neuropathic pain: redefinition and a grading system for clinical and research purposes	Neurology	36

## 4. Discussion

### 4.1. General information

This bibliometric analysis reveals that research on NP biomarkers is a field undergoing rapid expansion, with a consistent growth in annual publications over the past 2 decades. This surge is likely propelled by concurrent technological revolutions. The maturation of high-throughput technologies like sequencing, mass spectrometry, and protein microarrays has dramatically accelerated biomarker discovery.^[5,6]^ These innovative tools have enabled highly efficient, sensitive and cost-effective detection of target genes and proteins, expediting the discovery of novel biomarkers and elucidating their functions in complex biological processes.^[7,8]^ While advances in bioinformatics have provided the computational power to unearth meaningful signals from complex datasets.^[9–11]^

Our findings show this research growth is geographically concentrated, led by a strong contingent of authors and institutions from the United States and European countries. In contrast, China presents a distinct profile of high publication volume paired with a lower relative citation impact. This “citation gap” is a complex phenomenon observed in many emerging scientific fields. While it may reflect differences in research focus or international collaboration levels, it could also be influenced by factors such as language barriers or the natural time lag required for a large volume of recent publications to accrue citations. Attributing this gap to a single cause like “limited innovation” would be an oversimplification of the intricate dynamics of global scientific communication.

The scholarly dialogue within the field is channeled through a core set of high-impact journals. The prominence of publications in basic science journals like the International Journal of Molecular Sciences alongside clinically-oriented journals such as Pain underscores the field’s dual priorities. This landscape suggests a research ecosystem actively working to bridge the gap between foundational molecular discoveries and their application to clinical challenges, a key theme we will explore in the following analysis of research keywords.

### 4.2. Research hotspots and thematic evolution: from mechanism to technology

The keyword analysis provides a dynamic map of the field’s intellectual evolution, revealing a clear and decisive shift from foundational mechanistic studies toward technology-driven translational research. This evolution is defined by 2 parallel narratives. On one hand, enduring hotspots like “diabetic neuropathy” and “oxidative stress” underscore a sustained, decades-long commitment to unraveling the core pathophysiology of NP. On the other hand, the recent and powerful citation bursts for keywords such as “DTI,” “machine learning,” and “neurofilament light chain” signal a turn towards leveraging advanced neuroimaging, computational methods, and sensitive molecular diagnostics to solve clinical problems.

This overarching transition from basic science to clinical application is structured across the 5 major research clusters identified in our analysis: molecular mechanisms, clinical biomarker insights, biomarkers in neurodegenerative diseases, chemotherapy-induced neuropathy, and biomarker discovery via brain alterations. In the following sections, we will dissect each cluster to explore the specific trends and discoveries that characterize the modern landscape of NP biomarker research.

### 4.3. Red cluster: unraveling the molecular mechanisms of NP

The red cluster forms the foundational bedrock of the entire NP biomarker research landscape, centered on dissecting core pathological processes. The high frequency and centrality of fundamental biological keywords – such as “up-regulation,” “down-regulation,” “gene expression,” “central sensitization,” “astrocytes,” and “microglia” and “receptor” – confirm that a primary, sustained effort in the field has been to decipher the basic molecular grammar of NP. This focus is not abstract; it is tied to specific, widely-investigated hypotheses.

According to Hiroshi Ueda, nerve injury triggers the upregulation of several critical components in sensory neurons, including the Ca(v)α2δ-1 channel subunit, Na(v)1.3 sodium channel, bradykinin B1 receptor, and transient receptor potential vanilloid subfamily 1 (TRPV1) receptor. This upregulation leads to hypersensitivity in peripheral neurons, resulting in symptoms such as spontaneous pain, heat hyperalgesia, and cold allodynia.^[12]^ The central sensitization mechanism proposes that substances released by damaged peripheral neurons stimulate undamaged neurons, thereby increasing the excitability of dorsal horn spinal neurons. This heightened excitability manifests as tactile sensations being perceived as painful and involves processes such as N-Methyl-D-Aspartate (NMDA) receptor-dependent synaptic plasticity and activation of astrocytes and microglia.^[13,14]^ In addition, the prominence of “rat model” and “nerve injury” as core keywords within this cluster highlights the field’s deep reliance on preclinical models to dissect these complex pathways and test potential therapeutic interventions.^[12]^

Collectively, the keywords in this cluster paint a clear picture: the dominant strategy in NP biomarker research over the past 2 decades has been a “bottom-up” approach. This research has focused intensely on elucidating fundamental biological dysfunctions, establishing the essential knowledge base required before reliable and sensitive biomarkers can be identified and validated for clinical use.

### 4.4. Green cluster: bridging discovery to clinical application

If the red cluster represents the foundational “how” of NP pathophysiology, the green cluster embodies the translational “so what?” – the critical effort to connect molecular findings to tangible clinical outcomes. The high frequency of epidemiological and clinical research keywords such as “severity,” “risk factors,” “progression” “prevalence,” “population,” and “association” signifies a crucial focus on human-centered studies. This vocabulary is the language of cohort studies and clinical validation, where the goal shifts from discovery to rigorously testing a biomarker’s utility in real-world patient populations, as defined by regulatory bodies like the U.S. FDA.^[15]^

The core question in this cluster evolves from “Is this biomarker present?” to “Does this biomarker robustly predict disease onset, monitor progression, or forecast treatment response?” Our analysis shows this theme is not just an abstract goal but a vibrant area of research, substantiated by highly-cited studies that directly align with the keywords of this cluster. For example: Lorena et al^[16]^ investigated serum neurofilament light chain levels, incorporating 1-year follow-up data, in 98 Guillain–Barré syndrome patients and 53 healthy controls. Multivariate regression analysis revealed that elevated baseline serum NfL levels independently predict poor prognosis, confirming its role as a prognostic biomarker. The 2017 KORA F4/FF4 study^[17]^ identified soluble intercellular adhesion molecule-1 (sICAM-1) and interleukin-1 receptor antagonist (IL-1RA) as contributors to the progression of diabetic sensorimotor polyneuropathy (DSPN).

Therefore, this green cluster functions as the translational engine of the field. It highlights the challenging but essential research dedicated to bridging the gap between basic science discoveries and the development of clinically actionable tools that can guide personalized medicine and ultimately improve patient care.

### 4.5. The rise of disease-specific and pan-neuronal biomarkers

The blue and yellow clusters shift the focus from general mechanisms to the application of biomarker strategies in specific, high-prevalence neuropathies. The yellow cluster, defined by keywords like “quality of life,” “paclitaxel,” “neurotoxicity,” “chemotherapy” and “cancer” clearly delineates the major research effort into chemotherapy-induced peripheral neuropathy. The blue cluster captures a broader range of conditions, including the most common cause of NP, “diabetic peripheral neuropathy” (DPN), alongside a host of neurodegenerative diseases such as “amyotrophic lateral sclerosis” and “multiple sclerosis.”

A powerful trend that cuts across these disease-specific silos is the search for a universal marker of neuronal injury. Our analysis identified “neurofilament light chain” (NfL) as a key keyword with a strong and recent citation burst, and the literature provides a clear explanation for its emergence. As a direct indicator of axonal damage, elevated NfL levels are not confined to a single pathology. Studies have validated its utility as a prognostic and monitoring tool across a striking range of neuropathies, including DPN,^[18–21]^ Guillain-Barré syndrome,^[22,23]^ and chronic inflammatory demyelinating polyneuropathy.^[24]^ This broad applicability explains its powerful emergence as a “pan-neuronal” biomarker in our analysis.

However, the success of a general marker like NfL also highlights a core challenge in the field. While NfL effectively signals that nerve damage is occurring, it often lacks specificity for the underlying cause (e.g., metabolic, toxic, or autoimmune). This limitation underscores why research continues in parallel on more disease-specific biomarkers, such as the unique inflammatory markers identified in DPN or the distinct mechanistic pathways in chemotherapy-induced peripheral neuropathy. This dual approach – searching for both a universal damage indicator and highly specific causal markers – represents a key strategic tension that currently defines the frontier of NP biomarker research.

### 4.6. Purple cluster: the turn to brain-based biomarkers and neuroimaging

The purple cluster signifies a pivotal shift in biomarker discovery, moving beyond peripheral molecular markers to investigate the brain’s own structural and functional alterations in response to chronic pain. This is arguably the most forward-looking research frontier identified in our analysis, a conclusion strongly supported by the powerful and recent citation bursts for keywords such as “DTI” and “machine learning.” The rise of these keywords reflects a growing consensus that chronic pain is a neurological condition that actively remodels the brain, and that these changes can be captured by advanced neuroimaging.

This research effort is multifaceted. On the functional side, studies using fMRI have successfully identified changes in brain activation and resting-state functional connectivity that correlate with pain and analgesic effects. For instance, Endo et al^[25]^ identified activation in contralateral primary somatosensory cortex in a rat model using functional MRI (fMRI), recognizing this activation as a hallmark of NP. Similarly, Weizman et al^[26]^ conducted a randomized controlled trial involving 15 patients with chronic radicular NP and reported that reduced functional connectivity between the anterior cingulate cortex and the sensorimotor cortex was strongly linked to analgesic effects. Individual pain relief was further associated with decreased network connectivity in the dorsolateral prefrontal cortex (DLPFC), suggesting that baseline functional connectivity across brain regions could potentially serve as a predictive biomarker for pain relief outcomes.

More recently, as our keyword analysis suggests, machine learning models are being applied to this complex data to identify robust predictive signatures for chronic pain assessment. On the structural side, the emergence of DTI as a research hotspot is validated by studies using it to assess the integrity of white matter tracts and confirm anatomical changes in pain-processing pathways. For instance, Hadjipavlou et al^[27]^ used DTI to confirm anatomical connections between cortical and brainstem pain-processing regions. Behler et al^[28]^ proposed the use of machine learning models to analyze multi-modal DTI datasets to enable a comprehensive assessment of neuropathological characteristics, aiding in the development of neuroimaging biomarkers for clinical diagnosis.

The convergence of multi-modal neuroimaging with artificial intelligence, as mapped by this cluster’s keyword trends, represents a major strategic pivot in the field. The goal is no longer just to observe pain-related brain activity, but to identify objective, reliable, and noninvasive neuroimaging biomarkers that can be used for diagnosis, patient stratification, and predicting treatment outcomes, thereby moving the field closer to a truly brain-based approach to precision pain medicine.

### 4.7. Current challenges and future trends

Our bibliometric analysis of the NP biomarker landscape does more than map its history; it illuminates the critical challenges and strategic imperatives for its future. Two primary challenges emerge directly from the intellectual structure revealed by our analysis:

First, our data suggests a significant bottleneck in clinical translation. The vast and foundational “molecular mechanisms” cluster (Red) dwarfs the “clinical insights” cluster (Green), which focuses on validation in patient populations. This quantitative disparity indicates that while biomarker discovery is robust, the pipeline for rigorously translating these discoveries into standardized, cost-effective, and clinically validated tools is constricted. Overcoming this will require a concerted focus on large-scale, multicenter prospective studies – the very type of research represented by the smaller green cluster.

Second, the existence of distinct and semi-isolated research clusters – molecular (red), neuroimaging (purple), and disease-specific (blue/yellow) – highlights a degree of fragmentation in the field. This “siloed” approach, while effective for deep investigation, inherently limits the utility of any single biomarker type in capturing the full heterogeneity of NP. The clear future direction, therefore, is the integration of these parallel streams. A multi-modal strategy, combining genetic, proteomic, inflammatory, and neuroimaging markers, is essential for developing the next generation of diagnostic panels that reflect the multi-faceted nature of the disease.

In essence, this study provides a data-driven roadmap. Future progress will depend less on digging deeper into existing research silos and more on building bridges between them. Fostering collaboration between basic scientists, neuroimagers, and clinical trialists is the key to accelerating the development of robust, multi-faceted biomarker panels that can genuinely improve the lives of patients with NP.

## 5. Limitations

This study has several limitations that should be acknowledged. First, our analysis is based exclusively on the WoSCC. While extensive, this focus may exclude relevant publications indexed in other databases or gray literature, such as conference proceedings and dissertations. The scope of the retrieved literature is also inherently defined by the specific search keywords used; alternative terms could have yielded a different dataset. Second, citation-based metrics are subject to a significant time lag. Recently published articles have not had sufficient time to accrue citations, which may lead to an underestimation of the impact and emerging trends of the most current research. These factors should be considered when interpreting the presented map of the research landscape.

## 6. Conclusions

This bibliometric analysis provides a data-driven map of the NP biomarker research landscape, charting its rapid evolution over the past 2 decades. The field has been led by key contributors in the United States and Europe, with journals like *Pain* serving as central platforms for high-impact discourse. Our analysis reveals a field defined by a clear intellectual trajectory – from a deep foundation in molecular mechanisms toward an emerging frontier of technology-driven, brain-based biomarkers.

Despite this progress, our mapping of the research structure highlights a critical and persistent gap between basic discovery and clinical application. The very existence of distinct research silos underscores the primary challenge ahead: the need for integration. Future efforts must therefore prioritize building bridges between these research islands – fostering interdisciplinary collaboration between basic scientists, neuroimagers, and clinical trialists.

## Acknowledgments

All authors wrote the manuscript and approved the final version of the manuscript.

## Author contributions

**Conceptualization:** Zihao Zhang, Jia Ouyang, Ruen Liu.

**Data curation:** Zihao Zhang, Gaoquan Lv, Xin Chang, Yuepeng Wang.

**Formal analysis:** Zihao Zhang, Qingpei Hao, Gaoquan Lv, Shijun Peng, Tao Wang, Xin Chang, Yuepeng Wang.

**Funding acquisition:** Jia Ouyang, Ruen Liu.

**Investigation:** Zihao Zhang, Qingpei Hao, Shijun Peng, Xin Chang, Jia Ouyang.

**Methodology:** Zihao Zhang, Qingpei Hao, Gaoquan Lv, Shijun Peng, Tao Wang, Xin Chang, Yuepeng Wang.

**Project administration:** Zihao Zhang, Gaoquan Lv, Tao Wang, Xin Chang.

**Resources:** Qingpei Hao, Shijun Peng, Xin Chang, Yuepeng Wang, Ruen Liu.

**Software:** Zihao Zhang, Qingpei Hao, Gaoquan Lv, Shijun Peng, Yuepeng Wang.

**Supervision:** Gaoquan Lv, Shijun Peng, Tao Wang, Jia Ouyang, Ruen Liu.

**Validation:** Shijun Peng, Tao Wang, Xin Chang, Jia Ouyang, Ruen Liu.

**Visualization:** Qingpei Hao, Tao Wang, Jia Ouyang.

**Writing – original draft:** Zihao Zhang, Qingpei Hao, Gaoquan Lv.

**Writing – review & editing:** Shijun Peng, Tao Wang, Xin Chang, Yuepeng Wang, Jia Ouyang, Ruen Liu.
